# Antioxidant activity, nutritional and physicochemical characteristics, and toxicity of minimally refined brown sugar and other sugars

**DOI:** 10.1002/fsn3.1803

**Published:** 2020-07-31

**Authors:** Azrina Azlan, Hock Eng Khoo, Azliana Abu Bakar Sajak, Noor Atiqah Aizan Abdul Kadir, Barakatun Nisak Mohd Yusof, Zhafarina Mahmood, Sharmin Sultana

**Affiliations:** ^1^ Department of Nutrition & Dietetics Faculty of Medicine & Health Sciences Universiti Putra Malaysia Serdang Selangor Malaysia; ^2^ Research Centre of Excellence for Nutrition and Non‐Communicable Diseases Faculty of Medicine and Health Sciences Universiti Putra Malaysia UPM Serdang Serdang Selangor Malaysia; ^3^ Halal Products Research Institute Universiti Putra Malaysia UPM Serdang Serdang Selangor Malaysia; ^4^ Central Sugars Refinery Sdn. Bhd. Shah Alam Selangor Malaysia

**Keywords:** antioxidant properties, bioactive compounds, food safety, health, phenolic compounds, sugar

## Abstract

Minimally refined brown sugar (MRBS) is a brown sugar derived from sugarcane that has a low glycemic index. This study aimed to determine and compare the antioxidant contents and nutritional and physicochemical properties of MRBS, refined sugar (RS), and brown sugar (BS). In addition, the toxicity of these sugars was evaluated via in vitro cytotoxicity method and by using a zebrafish model. Results showed that MRBS was better than the two other sugars because it has a lower moisture content and higher ash content. The contents of potassium and manganese of MRBS were higher than those of the two other sugars. Surprisingly, MRBS also contained selenium, which was not detected in RS and BS. The major phenolics in MRBS are 4‐hydroxybenzoic acid, chlorogenic acid, protocatechuic acid, trans‐Ferulic acid, and apigenin. All sugar solutions and their antioxidant‐containing extracts were not cytotoxic to 3T3‐L1 adipocytes.

## Graphical abstract

Determination of antioxidant and toxicity in MRBS using in vitro cytotoxicity method and zebrafish model. Assessment of antioxidant activities of phenolic compound by DPPH radical scavenging assay. Illustration of the nutritional differences between refined sugar, brown sugar and minimally refined brown sugar.

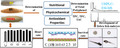

**Abbreviations**
BS: brown sugar
HPLC: high‐performance liquid chromatography
MRBS: minimally refined brown sugar
RS: refined sugar
TAC: total anthocyanin content
TFC: total flavonoids content
TPC: total phenolic content

## 1 INTRODUCTION

Cane sugar is an important foodstuff traded worldwide. Its consumption remains high because of its gustative, nutritional, and preservative properties that make it an essential nutrient (Payet, Sing, & Smadja, 2005). Cane sugar is a natural sweetener rich in sucrose and contains modest amounts of phytochemicals (Godshall, Vercellotti, & Triche, 2002). The predominant phytochemicals in sugarcane juice are phenolic acids (hydroxycinnamic acid, caffeic acid, and sinapic acid) and polyphenols and flavonoids (apigenin, luteolin, and tricin derivatives) (Duarte‐Almeida, Novoa, Linares, Lajolo, & Genovese, 2006; Duarte‐Almeida, Salatino, Genovese, & Lajolo, 2011). Traditional white cane sugars consist of nearly 99% of sucrose (Seguí, 2015), whereas brown cane sugars are composed of 88%–93% of sucrose and characterized by an exquisite flavor and odor (Payet et al., 2005). Cane sugar typically contains numerous nutrients and fibers. Their content depends on procedures employed to extract juices. These procedures tend to reduce their sugar content and deteriorate natural fibers and other ingredients. Sugars, such as brown sugar (BS) and refined sugar (RS), are usually sequentially processed: washing, extraction, purification, crystallization, drying, and packaging. Owing to it is the high purity of RS, its nutritional value low, and thus, it provides empty calories (Lee et al., 2018).

By contrast, BS has higher amounts of phenolics than RS. For instance, the total phenolic content of cane BS ranges from 108.1 µg to 259.6 µg gallic acid equivalent (GAE)/g sample (Payet et al., 2005). Phytochemicals play an important role in maintaining physical health. However, the phytochemicals of BS are low. Aside from conferring sweet taste and extra energy, the phytochemicals in cane sugar help improve health and reduce the risk of metabolic diseases (Guimarães et al., 2007; Payet et al., 2005).

Minimally refined brown sugar (MRBS) is one of the most important natural brown sugars. MRBS is directly produced from food grade sugar mills by a sequential process involving washing of raw materials, extraction, minimal refining, crystallization, drying, and packaging according to WHO standards. Hence, MRBS is not processed as much and less refined unlike other sugars; moreover, MRBS derived from sugarcane is not genetically modified (Jaffé, 2015). The fewer processes involved in producing MRBS retain some naturally occurring trace minerals (such as calcium, magnesium, and potassium), vitamins, amino acids, antioxidants, and phytochemicals (Lee et al., 2018). Given that health professionals and officials recommend increasing the intake of foods rich in antioxidants, substituting RS with MRBS may offer a potential extra source of antioxidants.

In general, all types of cane BS are nontoxic sweetener widely consumed worldwide. Interest in polyphenols, including flavonoids and phenolic acids, has considerably increased because several studies have demonstrated that the toxic effects of phenolic compounds depend on phenolic concentration (Guimarães et al., 2007; Payet, Sing, & A., Smadja, J, 2006; Valli et al., 2012). Numerous studies have evaluated the potential toxicity of natural sweeteners, such as honey, molasses, and syrups (Guimarães et al., 2007; Payet et al., 2006; Seguí, Calabuig‐Jiménez, Betoret, & Fito, 2015; Valli et al., 2012). MRBS has been recently commercialized in Malaysia because of its antioxidant content. However, the potential health effects of cane MRBS as a substitute for RS on food have not been assessed. Moreover, its toxicity has not been evaluated yet. In this study, we investigated the antioxidant activity, nutritional and physicochemical characteristics, vitamin and mineral contents, and toxic effects of MRBS via in vitro chemical and biological assays. We evaluated the cytotoxic effects of these sugars via in vitro cytotoxicity method and a zebrafish model (van Eyk, 2015; Valli et al., 2012). Finally, we compared MRBS with commercial BS and sugars in terms of the said parameters.

## 2 MATERIALS AND METHODS

### 2.1 Preparation of samples

RS, commercial BS, and MRBS were obtained in triplicate from the Central Sugar Refinery Sdn Bhd, Malaysia. All sugar samples (5.0 kg each) were stored at room temperature (25°C) until use. All chemicals, standards, and solvents of analytical grade were purchased from Sigma‐Aldrich Company (Germany).

### 2.2 Extraction of polyphenols

Polyphenols were extracted following the method of Chen, Zhao, and Yu (2015) with some modifications to obtain extracts of good quality. Solid samples were ground into powder and thoroughly homogenized with liquid nitrogen. Polyphenols were extracted by mixing 1.0 g of sugar samples with 10 ml of acidified ethanolic solution (1.6 M HCl in 60% ethanol, v/v). The mixtures were vigorously stirred for 1 min and then ultrasonicated using a Powersonic 405 ultrasonic homogenizer for 20 min (Hwashin Technology Co). The solvent was removed by a rotary vacuum evaporator at 45°C, and the aqueous extracts obtained were used to determine antioxidants via in vitro assays and to evaluate the cytotoxic effects of the sugar extracts.

### 2.3 Determination of fats, proteins, and carbohydrates

Proximate compositions of the sugar samples were determined following AOAC methods (AOAC, 2012). In accordance with AOAC method 991.36, fats were extracted using petroleum ether as the extraction solvent in a Soxhlet extractor. The sugar samples were hydrolyzed according to the Weibull–Stoldt method to extract fats. The sugar samples (100 g) were hydrolyzed using 100 ml of 3 M HCl for 1 hr under reflux.

Protein contents of the sugar samples were determined following the AOAC method 981.10 and the Kjeldahl method. Total nitrogen contents were determined using a Kjeltec automatic analyzer (Protein‐Gerhardt VAP50 Vapodest, Konigswinter, Germany). The recovery rate of the Kjeldahl method was 80%–120%.

Carbohydrate contents were determined as total sugar content on the basis of the AOAC method 982.14 with some modifications (AOAC, 2012). Carbohydrate content was calculated as.

$$\%\text{component}=\left(R/R\prime \right)\times \left(C/W\right)\times V\times 100,$$

where *R* and *R*′ are the peak heights and peak area of sample or standard, respectively; *V* is the volume of sample; *W* is the weight of sample; and *C* is the concentration of sugar standard (g/ml). Each experiment was performed in triplicate.

### 2.4 Determination of total dietary fibers, moisture contents, ashes, and minerals

Moisture contents were determined by placing a 2 g sugar sample in a drying oven at 105 ± 2°C until a constant weight was achieved following the AOAC standard method no. 991.43 (dietary fiber) (AOAC, 2012). Ash contents were determined by placing a 2 g sugar sample in a crucible and heating it in a muffle furnace to 550°C for 6 hr according to the AOAC method no. 923.03 (AOAC, 2012).

Mineral contents 2 g sugar samples were determined according to the AOAC method nos. 968.08 and 965.09 (AOAC, 2012). Maximum absorption of Ca, Fe, K, Mg, Zn, Co, and Na was set at 422.7, 248.3, 766.5, 285.2, 213.9, 324.7, and 589.0, respectively. Standard solutions of all minerals were prepared for standard calibration curves at four different concentrations of 1–4, 1–7, 0.1–0.8, and 1–5 ppm for calcium, iron, sodium, and copper, respectively, as well as 0.1–1.5 ppm for potassium, magnesium, and zinc (*R* ^2^ = 0.99). All mineral standards had a recovery of 73%–111%. Each experiment was performed in triplicate.

### 2.5 Determination of B vitamins

B vitamins were determined on the basis of methods described by Ekinci and Kadakal (2005) and Kamman et al. (1980) with method optimization. Vitamin contents of diluted sugar solutions were analyzed via high‐performance liquid chromatography (HPLC) with a C18 column (150 mm × 4.6 mm, I.D. 5 µm) (Milford, USA). The mobile phase used to separate other B vitamins was a mixture of water, methanol, and glacial acetic acid at a ratio of 73:27:1 containing 1.4 mg/ml sodium hexane‐1‐sulfonate. The injection volume was 20 µl with a flow rate of 0.8 ml/min. A UV detector (Maryland, USA) was set at 204 nm for vitamin B5 and at 275 nm for the other B vitamins. The total run time of HPLC analysis was set to 20 min.

The standards used for B vitamins were thiamin, riboflavin, niacin, pantothenic acid, pyridoxine, and folic acid. Different concentrations (5, 10, 25, 50, and 100 ppm) of the standards were prepared for standard calibration.

### 2.6 Estimation of total phenolic, total flavonoid, and total anthocyanin contents

Total phenolic contents (TPC) were estimated using the Folin–Ciocalteu method described by Wu et al. (2006) with slight modifications. An aliquot of 0.125 ml of the sugar solution (25 mg/ml) was added with 0.5 ml of Folin–Ciocalteu reagent in a test tube. All tubes were vortexed for 15 s and then incubated at room temperature (25°C). After 6 min, 1.25 ml of 7.5% sodium carbonate solution was added to the mixture and topped up to 10 ml with distilled water. Absorbance was measured at 760 nm by using a Hewlett Packard UV‐vis spectrophotometer (Palo Alto, CA). TPC was calculated on the basis of a standard curve of gallic acid solutions at five concentrations ranging from 0.05 to 2.0 mg/ml. Results were expressed as gallic acid equivalents (mg GAE/100 g).

Total flavonoid contents (TFC) were determined via the aluminum chloride colorimetric method (Seguí et al., 2015). In brief, 1.5 ml of the extracts was mixed with 1.5 ml of aluminum chloride solution (2% w/v in methanol). The mixture was vigorously shaken and allowed to react for 10 min. Absorbance of the mixture at 368 nm was measured, and flavonoid content was expressed in milligram of quercetin equivalents per gram of fresh weight.

Total anthocyanin contents were estimated via the spectrophotometric pH differential method (Wu et al., 2006). The solutions (25 mg/ml) were thoroughly mixed with 0.025 M potassium chloride at pH 1.0. Absorbance of the mixture at 510 and 700 nm was measured using distilled water. The mixtures were then combined with 0.4 M sodium acetate buffer at pH 4.5, and their absorbance was measured at the same wavelength. Absorbance was measured as follows:

$$\text{Absorbance}={\left(A510‐A700\right)}_{\text{pH}1.0}‐{\left(A510‐A700\right)}_{\text{pH}4.5}.$$

Total anthocyanin contents were calculated as the total monomeric anthocyanins:

$$\text{TAC}\left(\text{mg}/100g\right)=\left(A\times \text{MW}\times \text{DF}\times 1000\right)/\left(\varepsilon \times 1\right)/100g,$$

where *A* is the absorbance of diluted sugar solutions, MW is the molecular weight of cyanidin‐3‐galactoside (484.84), DF is dilution factor, and ε is equal to 34,300 M/cm. Each experiment was performed in triplicate.

### 2.7 Determination of phenolic compounds

Phenolic compounds were separated via the method described by Zakaria et al. (2019) with some modifications by using a Hypersil GOLD column (1.9 µm, 2.1 mm × 100 mm) (Thermo Scientific). The stationary phase was attached to a Dionex Ultimate 3000 Series UHPLC (Thermo Scientific). Phenolic compounds were separated on the basis of a gradient elution of formic acid in water (A) and formic acid in acetonitrile (B) at a flow rate of 0.4 ml/min and injection volume of 2.0 µl. Gradient run was set as follows: 5%–90% B for 5.0 min, 90%–90% B for 2 min, 90%–5% for 2 min, and 5%–5% for 3 min. Total run time of LCMS analysis was 12 min with a 3 min postrun time.

UV‐vis detection wavelengths were set at 254, 280, and 360 nm. Heated electrospray ionization conditions were set with capillary temperature of 320°C, spray voltage of 3,700 V, gas flow rate of 18 L/min, and nebulizer pressure of 45 psi. Mass spectrometry data were documented in negative ion mode. Mass spectrometry data were acquired within 50–750 *m/z* for both positive and negative ion modes. Data were analyzed using Xcalibur 2.2 software (Thermo Fisher Scientific, Waltham, MA). Phenolic compounds were quantified on the basis of standard calibration curves of phenolic acids (benzoic acid, 4‐hydroxybenzoic acid, caffeic acid, chlorogenic acid, p‐coumaric acid, trans‐Ferulic acid, protocatechuic acid, syringic acid, and vanillic acid) and flavonoids (apigenin, luteolin, tricin, and vanillin). These phenolic acid standards were obtained from Sigma‐Aldrich (M) Sdn Bhd (Selangor, Malaysia). The coefficient correlation values (*R* ^2^) of all standard calibration curves were higher than 0.9.

### 2.8 DPPH radical scavenging capacity assay

DPPH radical scavenging capacity assay was performed following the method of Wu et al. (2006) with slight modifications. In brief, an aliquot of 0.1 ml of 10 mg/ml sugar solutions was mixed with 2.0 ml of 0.6 mM DPPH radical solution in 80% ethanol. The tubes were vortexed for 15 s and allowed to stand at room temperature for 30 min in the dark. Absorbance of the mixture at 517 nm was measured using a UV‐vis spectrophotometer (SECOMAM SA, Alѐs, France). A blank of 80% ethanol was used for this assay. The control used was a DPPH radical solution without the test samples. Inhibition activity was calculated using the following equation:

$$\%\text{inhibition}=\left(\text{A control}‐\text{A sample}\right)/\text{A control}\times 100.$$

### 2.9 Ferric ion reducing antioxidant power assay

Antioxidant activity was estimated on the basis of ferric reducing antioxidant power (FRAP) assay described by Wu, Lin, Lin, Ken, and Wen (2007) and Pulido, Bravo, and Saura‐Calixto (2000) with some modifications. FRAP reagent was freshly prepared by mixing 2.5 ml of 10% trichloroacetic acid, 2.5 ml of 1% potassium ferricyanide, and 2.5 ml of 0.2 M phosphate buffer at pH 6.6. In brief, 2.5 ml of the FRAP reagent was added with 2.5 ml of distilled water, 0.5 ml of 1% iron (III) chloride, and 1.0 ml of 10 mg/ml sugar solutions and then incubated at 50°C for 30 min. Reagent blank was prepared by mixing the test samples without reagent. Absorbance of the reaction mixture was measured at 593 nm. Known concentrations (100–2000 µM) of iron (II) were used for calibration. FRAP values of the sugar solutions were calculated on the basis of standard calibration curves and expressed as µM Fe (II).

#### 2.9.1 Cytotoxicity of 3T3‐L1 cell lines

3T3‐L1, a preadipocyte cell line (ATCC), was obtained from the Institute of Biosciences, Universiti Putra Malaysia. The adipocytes were cultured and maintained in a complete growth medium containing high‐glucose DMEM supplemented with 10% fetal bovine serum and 100 U/mL penicillin–streptomycin in a 5% (CO_2_) incubator at 37°C (Castillo, González, & Moore‐Carrasco, 2019). Cytotoxicity was measured using the MTT assay (Bahuguna, Khan, Bajpai, & Kang, 2017). In brief, 3T3‐L1 cells were seeded at 2 × 10^3^ cells per well in a 96‐well plate containing 100 µl of complete growth medium per well. The plate was incubated in the CO_2_ incubator until the cells reached 70%–80% confluence. The medium was then removed and replaced with a freshly prepared medium containing 0.05–200 mg/ml of the sugar samples. The plates were incubated for 24, 48, and 72 hr before measuring sugar cytotoxicity via the MTT assay. Afterward, the medium (containing media and samples) was removed, and the plate was washed with phosphate buffer saline (PBS, pH 7.4) to remove dead cells and debris. Subsequently, a medium mixture containing 20 µl of MTT reagent (5 mg/ml in PBS) was prepared, and then, 100 µl of the medium was added to each well of the plate and wrapped with aluminum foil. The plate was then incubated at 37°C in the CO_2_ incubator for 44 hr until the purple formazan was visible under the microscope. The medium was then discarded, and the crystalized formazan was dissolved with 100 µl of dimethyl sulfoxide and swirled on a microplate shaker for 15 min. The dissolved formazan was measured using a microplate reader at 540 nm (Molecular Devices Corp., VERSAmax, and Sunnyvale, CA, USA). All experiments were run in three replicates on three different days.

### 2.10 Acute toxicity study (zebrafish model)

#### 2.10.1 Staging of fish embryos

**Figure 1:**
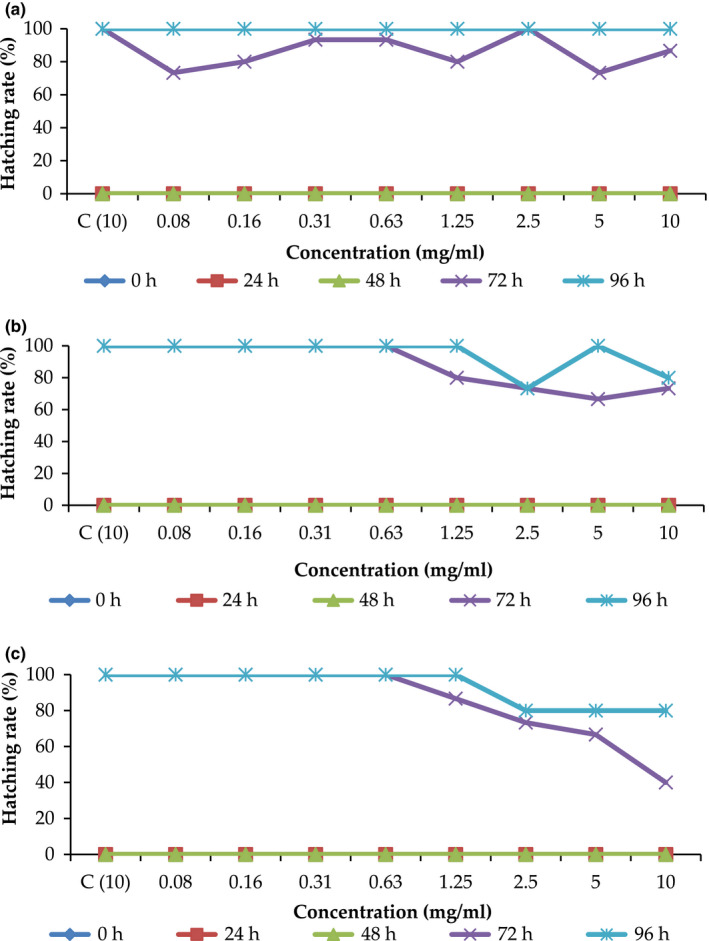
The development and morphology of zebrafish embryos. (A) Zygote – 0 hr, (B) segmentation period – 24 hr, (C) pharyngula period – 48 hr, (D) hatching period – 72 hr, and (E) larval period – 96 hr

The procedures for zebrafish acute toxicity experiments were adopted from Ding and Chen (2012) and Sata, Bakar, Ramlan, and Ibrahim (2016) with slight modifications. The health of zebrafish embryos was examined before performing the acute toxicity tests. In the tests, coagulated eggs were replaced with new healthy embryos (Figure 1).

#### 2.10.2 Exposure of embryos to sugar solutions

All embryos at 24 hr postfertilization (hpf) were cultivated in 96‐well cell culture plates by using a Pasteur pipette (Zhang, Lu, Gelinas, Ciruna, & Sun, 2011). The hatching, survival, and heart rates of all embryos were measured at each check‐point. Before treatment, the embryo media were discarded from the wells to ensure that the sugar solution was treated at the desired concentration.

Exposure tests were performed by treating the zebrafish embryos with different concentrations (0.08, 0.16, 0.31, 0.63, 1.25, 2.5, 5.0, and 10.0 mg/ml) of the samples at different durations (0, 24, 48, 72, and 96 hr). In brief, 100 µl of the sugar samples was dissolved at concentrations of 0.08–10.0 mg/ml with sterile deionized water. The solution was transferred into each well by using a pipette, and each well contained five embryos. Embryonic development was observed and recorded at 24 hr intervals. The hatching rate, scoliosis, and heartbeat (beats/min) of the embryos were recorded and analyzed at the end of the treatment (96 hr). Images of the morphology of zebrafish embryos were obtained using an inverted microscope with a built‐in camera (Hoage, Ding, & Xu, 2013; Ismail et al., 2017).

### 2.11 Statistical analysis

All analyses were conducted in triplicate (*n* = 3) for each sugar sample. Minitab version 18 was used for statistical analysis. Significant differences between the mean values of the samples were statistically analyzed using one‐way ANOVA coupled with LSD multiple comparisons.

## 3 RESULTS AND DISCUSSION

### 3.1 Proximate composition analysis

The proximate compositions of all sugar samples are presented in Table 1. Results showed that BS samples had the highest protein, dietary fiber, and moisture contents, but the samples had the lowest carbohydrate content at *p* < .05. MRBS samples had the highest ash content (0.09%). These findings were somewhat consistent with those of Lee et al. (2018), who reported that unrefined sugars have higher ash contents (0.02%–1.54%) than refined sugars. Previous studies suggested that the ash content of unrefined sugars should not exceed 2.2% because a high ash content is attributed to a high potassium content, which may impart an unpleasant taste and hinder sugar crystallization (Lee et al., 2018; Lopes & Borges, 2004). In the present study, the ash contents of the sugars (0.01%–0.09%) were below the level proposed by Lopes and Borges (2004). The moisture content of the tested sugars ranged from 0.1% to 0.15%. The moisture content of BS (0.15%) was significantly higher than that of RS (0.1%) and MRBS (0.11%). Variations in moisture content among sugars are due to differences in processing conditions (Jaffé, 2015). However, a high moisture content promotes crystal dissolution, biochemical degradation reactions, microbial deterioration, and cobblestone formation, all of which shorten the shelf life of sugars (Guerra & Mujica, 2010). However, the difference in energy content among the samples was not significant (at *p* > .05). Further assays revealed that RS had the lowest amounts of many nutrients, such as protein, ash, dietary fiber, and moisture. Aside from these nutrients, dietary fiber was not detected in RS samples. By contrast, RS samples had the highest carbohydrate content because white cane RS contains low to trace amounts of antioxidants (Phillips, Carlsen, & Blomhoff, 2009) and other nutrients besides sucrose. HPLC revealed that the sugar samples contained 97% sucrose as the main carbohydrate. However, glucose, fructose, and maltose were not detected. As shown in Table 1, RS contained the lowest amount of sucrose (96.57%), whereas MRBS and BS had slightly higher sucrose content (97.3%). The same amount of sucrose in MRBS and BS was probably due to the fact that both sugars are regular BS. The only difference between these sugars is their processing procedures. MRBS undergoes fewer refining processes than BS. Raw BS has about 92% of total sugar, of which 84.5% is sucrose (Jaffé, 2015). In the present study, the sucrose content of both MRBS and BS samples was higher than that of raw BS reported in the literature. This high amount of disaccharides in BS correlates well with the low GI compared. The influence of different raw materials and various manufacturing processes may explain the variations observed herein. In addition, mass accumulation of sucrose in sugar cane is hypothesized to be caused by the activities of certain enzymes in cane tissues (Rohwer & Botha, 2001). For instance, sucrose phosphorylase is known to catalyze sucrose synthesis from uridine diphosphate glucose (Cardini, Leloir, & Chiriboga, 1955). Therefore, BS and MRBS are supposed to retain diphosphate glucose because they undergo less treatment than RS (Valli et al., 2012).

**Table 1:** Proximate composition and physicochemical characteristics of sugar samples

Proximate composition			
Name of test	Refined	Brown	MRBS
Protein (g/100 g)*, a	0.05 ± 0.01^c^	0.12 ± 0.01^a^	0.08 ± 0.01^b^
Fat (g/100 g)	0.11 ± 0.02^a^	0.58 ± 0.31^a^	0.04 ± 0.01^a^
Carbohydrate (g/100 g)*, a	99.81 ± 0.03^a^	96.8 ± 0.12^c^	98.0 ± 0.04^b^
Glucose (g/100 g)	ND	ND	ND
Fructose (g/100 g)	ND	ND	ND
Sucrose (g/100 g)	96.57 ± 0.00b	97.28 ± 0.06a	97.31 ± 0.00a
Maltose (g/100 g)	ND	ND	ND
Ash (g/100 g)*, a	0.01 ± 0.00^c^	0.07 ± 0.01^b^	0.09 ± 0.01^a^
Dietary fiber (g/100 g)*, a	ND	2.38 ± 0.00^a^	1.67 ± 0.00^b^
Moisture (g/100 g)*, a	0.01 ± 0.00^c^	0.15 ± 0.00^a^	0.11 ± 0.00^b^
Energy (kcal/100 g)	400.43 ± 0.09^a^	402.04 ± 1.52^a^	399.39 ± 0.02^a^
*Physicochemical characteristics*			
Acidity (mEq/kg)	0.05 ± 0.00	0.05 ± 0.00	0.10 ± 0.00
Brix, % (10% solution)	50.8 ± 0.00	52.0 ± 0.00	52.3 ± 0.00
Color			
L	83.36 ± 0.16	59.38 ± 0.70	65.79 ± 0.88
a	0.56 ± 0.02	3.25 ± 0.02	2.93 ± 0.02
b	5.23 ± 0.02	16.28 ± 0.09	15.59 ± 0.11

Note

Data are shown as mean ± standard error.

* Significant difference at *p* < .01 (ANOVA); different lowercase superscript letters denote significant differences at *p* < .05 (LSD). ND: <0.01 g/100 g.

### 3.2 Physicochemical characteristics

Physicochemical characteristics, such as acidity, Brix, and color, were determined. Results showed that the physicochemical characteristics were significantly different among all sugar samples (Table 1). LSD post hoc test revealed that MRBS had the highest acidity and Brix values. MRBS also had moderate values of colors (L, a, and b). By contrast, RS had the lowest acidity, Brix values, and color (a and b) values. RS also had the highest L color value. BS had the lowest L color value, but its a and b color values were the highest among all sugar samples (Table 1).

A sugar sample with high acidity indicates that it has undergone fewer processing and refining steps. MRBS had the highest acidity, thus confirming that it was the least processed cane sugar. The higher acidity of MRBS than the two other sugar samples may confer it with some advantages for use as a natural sweetener. High acidity helps regulate the acidity of a juice or drink added with MRBS. MRBS had the highest Brix value; thus, it had the highest amount of glucose and sweeter than the two other sugars.

### 3.3 Determination of vitamins and minerals

Only B vitamins were determined because both BS and MRBS have minuscule amounts of B vitamins, such as riboflavin, niacin, B6, B5, and folate (Melodie Anne, 2018). However, HPLC analysis revealed that the concentrations of B vitamins were below the quantification limit. The amounts of B vitamins in the sugar samples were <0.02 mg/100 g, <0.01 mg/100, <0.3 mg/100, <0.2 mg/100, and < 0.01 µg/100 g for B1, B2, B3, B6, and B9, respectively. Moreover, the B vitamins in all sugar samples were in trace amounts. The literature supports the present findings that cane sugars and sugarcane juices have low to trace amounts of B complex vitamins, such as thiamine (B1), riboflavin (B2), niacin (B3), and pantothenic acid (B5).

Among the essential minerals determined in the sugar samples (Table 2), phosphorus was not detected. Only selenium (0.02 mg/kg sample) was detected in MRBS. MRBS had the highest level of potassium and magnesium (*p* < .01). RS had the lowest amounts of all essential minerals (Table 2). Mineral content can be correlated with ash content. Thus, given that MRBS had the highest ash content (0.09 mg/100 g), this sugar had the highest total mineral content. Similar findings in refined and unrefined sugars have been reported (Lee et al., 2018). Given that RS undergoes numerous processing steps, most of its nutrients, including minerals, are removed or lost because of heavy processing, polishing, and refining (Hansen, Harholt, Oikawa, & Scheller, 2012).

**Table 2:** Vitamin and Minerals content of sugar samples

	Refined	Brown	MRBS
Vitamin^*^, ^a^ content			
*Name of the Vitamin identified*			
B1 (mg/100 g)	<0.02	<0.02	<0.02
B2 (mg/100 g)	<0.01	<0.01	<0.01
B3 (mg/100 g)	<0.3	<0.3	<0.3
B6 (mg/100 g)	<0.2	<0.2	<0.2
B9 (µg/100 g)	<0.01	<0.01	<0.01
Minerals^*^, ^a^ content			
*Name of the Minerals identified*			
Ca (mg/kg)	1.755 ± 0.01	89.49 ± 0.01	49.48 ± 0.01
Fe (mg/kg)	ND	12.23 ± 0.01	0.03 ± 0.00
K (mg/kg)	16.15 ± 0.02	142.10 ± 0.01	327.11 ± 0.01
Na (mg/kg)	34.96 ± 0.01	47.81 ± 0.01	36.01 ± 0.01
Mg (mg/kg)	2.07 ± 0.01	55.21 ± 0.01	55.66 ± 0.01
Mn (mg/kg)	0.01 ± 0.00	0.28 ± 0.00	0.61 ± 0.01
P (mg/kg)	ND	ND	ND
Se (mg/kg)	ND	ND	0.02 ± 0.00
Cr (mg/kg)	0.9 ± 0.01	1.79 ± 0.07	0.91 ± 0.02

Note

Data are shown as mean ± standard error.

^a^ Significant difference at *p* < .01 (ANOVA coupled with LSD). *ND: <0.01 g/100 g.

The presence of selenium in MRBS makes it an ideal candidate for low GI sweetener with antioxidants. Other cane sugars may also exhibit contain high selenium content. Given that selenium is an insulin mimic (Zhu et al., 2013), MRBS is a good choice of sweetener for patients with diabetes. MRBS is also rich in potassium. Potassium has antihypertensive effects. A recent population‐based cross‐sectional study revealed that a low dietary intake of sodium increases the risk of diabetes (Sun et al., 2013).

### 3.4 Antioxidant contents of sugar samples

The polyphenol contents of BS and MRBS were determined following the Folin–Ciocalteu method. Results showed that MRBS (2.67 mg GAE/100 g sample) had the highest TPC, followed by BS (1.73 mg GAE/100 g sample) and RS (0.32 mg GAE/100 g) (Table 3). One‐way ANOVA was performed to verify this result. The TPC of MRBS was significantly higher than that of BS and RS. RS, light BS, dark BS, and raw BS have a TPC of 0.004, 0.38, 0.42, and 0.58 mg GAE/g, respectively (Seguí et al., 2015). The TPC of the studied RS was (0.32 mg GAE/g), which was higher than that of RS reported in the literature (0.004 mg GAE/g). Similarly, the TPC of BS (1.73 mg GAE/g) and MRBS (2.67 mg GAE/g) was also higher than that of BS reported in the literature (0.4–0.6 mg GAE/g).

**Table 3:** Antioxidants content and antioxidant activities of MRBS and other sugars

Antioxidant parameter	Refined	Brown	MRBS
TPC (mg GAE/g)	0.32 ± 0.03^c^	1.73 ± 0.01^b^	2.67 ± 0.01^a^
TFC (mg QE/g)	0.40 ± 0.01^c^	2.64 ± 0.38^b^	3.28 ± 0.01^a^
TAC (C3GE, mg/g)	ND	0.04 ± 0.01^a^	0.04 ± 0.01^a^
DPPH (% inhibition)	89.70 ± 0.69^b^	88.11 ± 1.24^b^	92.48 ± 0.98^a^
FRAP (mM Fe^2+^/g)	1.16 ± 0.08^c^	3.08 ± 0.09^b^	5.53 ± 0.02^a^

Note

Different lowercase superscript letters (a, b) show a significant different between the sugar samples.

Abbreviations: C3GE: cyanidin‐3‐galactoside equivalent; FRAP: ferric ion reducing antioxidant power; GAE: gallic acid equivalent; MRBS: minimally refined brown sugar; ND: not detected; TAC: total anthocyanin content; TFC: total flavonoid content; TPC: total phenolic content.

However, the TPC of RS reported by Seguí et al. (2015) was quite low (~0.022 mg quercetin equivalent/g sample). Other studies have reported a wider range of TPC in BS (0.37 mg GAE/g, Nayaka et al. 2009; 0.1–0.41 mg GAE/g, Payet et al., 2005). Nayaka et al. (2009) obtained a high TPC value of 3.83 mg GAE/100 g sample in jaggery. These findings demonstrated that RS and BS contain fair amounts of polyphenols. Moreover, variations in TPC are not surprising because phenolic compounds are strongly involved in the color formation of sugar products (Payet et al., 2005). In addition, several studies have demonstrated that certain sugars, such as sucrose and glucose, do not remarkably react at room temperature with Folin reagent on a molar basis, but they may interfere with the test result by enhancing the development of blue color (Payet et al., 2005). This interference may be the cause of low TPC values of RS reported in the literature. Nevertheless, the presence of high sucrose content in the samples might have substantially affected the TPC results of BS and MRBS, which contain approximately 97% sucrose.

### 3.5 Identification and quantification of phenolic acids

**Figure 2:**
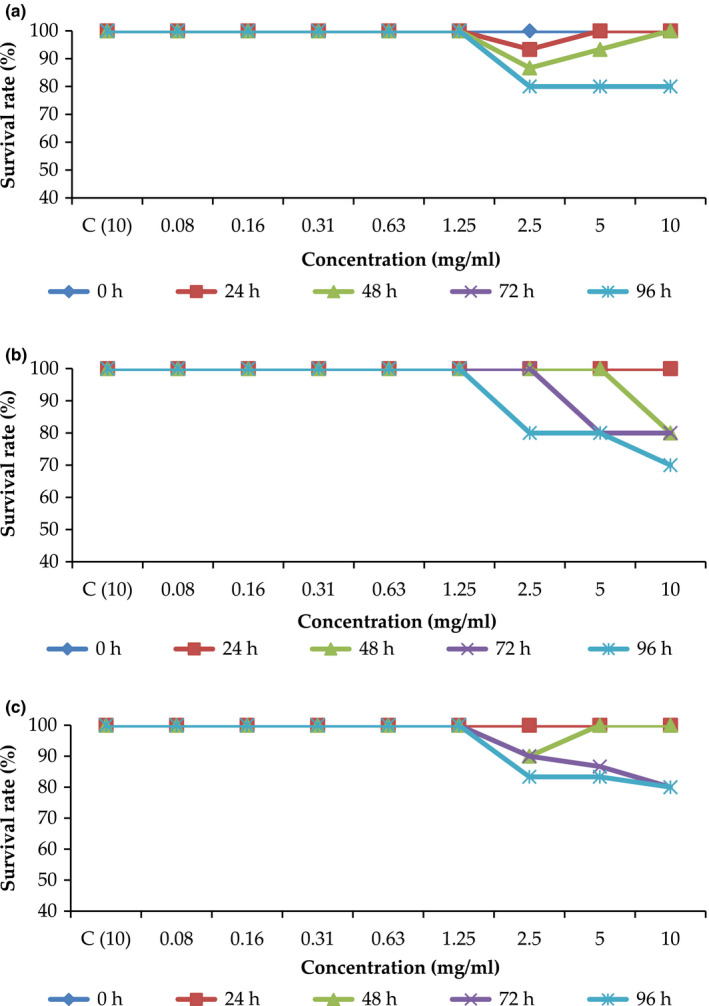
HPLC chromatogram and TIC of refined sugar (A), brown sugar (B), and minimally refined brown sugar (C) samples. The major peaks were tentatively identified as 2,3,5,6‐tetrachloro‐4‐(4‐phenylhepta‐1,2,5,6‐tetraen‐4‐yl)phenol (A), the fragment ions (A1‐A5) were determined as m/z 367, 359, 337, 323, 229, and 213, respectively

The phenolic compounds of the sugar samples were determined via UHPLC‐ESI‐MS. HPLC chromatograms and TICs of the sugar samples are shown in Figure 2. Except for 4‐hydroxybenzoic acid, protocatechuic acid, and chlorogenic acid, the phenolic acids among the sugar samples were significantly different. As shown in Table 4, these three phenolic acids were determined in trace amounts (<1.0 µg/g sample). Trace amounts of trans‐Ferulic acid (1.15 ± 0.75 µg/g) were determined only in BS samples. By contrast, caffeic acids were found only in MRBS (1.93 ± 1.84 µg/g) and BS (7.83 ± 3.74 µg/g) samples. Moreover, the sugar samples had benzoic acid content ranging from 4.771 µg/g sample to 8.403 µg/g sample.

**Table 4:** Phenolic acids content of sugar samples

	Molecular formula	RT (min)	[M–H]^–^	Refined	Brown	MRBS
*Phenolic acids* ^a^						
Benzoic acid*	C_7_H_6_O_2_	5.92	121	7.21 ± 1.33^a^	8.40 ± 3.12^a^	4.77 ± 1.34^a^
4‐Hydroxybenzoic acid	C_7_H_6_O_3_	2.83	137	Trace	Trace	Trace
Caffeic acid*	C_9_H_8_O_4_	3.77	179	Trace	1.93 ± 1.84^a^	7.83 ± 3.74^a^
Chlorogenic acid	C_16_H_18_O_9_	3.06	353	Trace	Trace	Trace
p‐Coumaric acid*	C_9_H_8_O_3_	4.64	163	17.56 ± 5.07^a^	19.86 ± 3.13^a^	6.47 ± 3.50^a^
trans‐Ferulic acid	C_10_H_10_O_4_	5.23	193	Trace	1.15 ± 0.75	Trace
Protocatechuic acid	C_7_H_6_O_4_	1.92	153	Trace	Trace	Trace
Syringic acid*	C_9_H_10_O_5_	3.75	197	9.39 ± 2.17^a^	2.14 ± 0.76^a^	17.32 ± 6.67^a^
Vanillic acid	C_8_H_8_O_4_	3.50	167	7.09 ± 2.23^a^	6.32 ± 3.62^a^	4.81 ± 2.1^a^
Total				41.25	39.80	41.20
*Flavonoids* ^a^						
Apigenin	C_15_H_10_O_5_	9.36	269	<0.2	<0.3	<0.3
Luteolin	C_15_H_10_O_6_	8.18	285	<0.2	<0.2	<0.1
Tricin	C_17_H_14_O_7_	9.61	329	<0.2	<0.4	<0.3
						

Note

Data are shown as mean ± standard error (µg/g sample).

^a^ The values of flavonoids in the sugar samples are not quantifiable due to concentrations are below the quantification limit. Trace (<1.0 µg/g sample—below the quantification limit).

* Significant difference at *p* < .01 (ANOVA); different lowercase superscript letters denote significant differences at *p* < .05 (LSD).

The phenolic acids that have antioxidative effects are caffeic acid, p‐coumaric acid, ferulic acid, syringic acid, vanillic acid, and chlorogenic acid. The amounts of these phenolic acids in RS samples were moderately low (Payet et al., 2006). By contrast, the amount of p‐coumaric acid in RS sample was 17.564 µg/g sample, comparable with that of other sugar samples. The amounts of syringic acid and vanillic acid in RS were also comparable with those of BS and MRBS. Although MRBS had trace amounts of chlorogenic acid and 4‐hydroxybenzoic acid, MRBS had the highest concentrations of these phenolic acids (data not shown).

**Figure 3:**
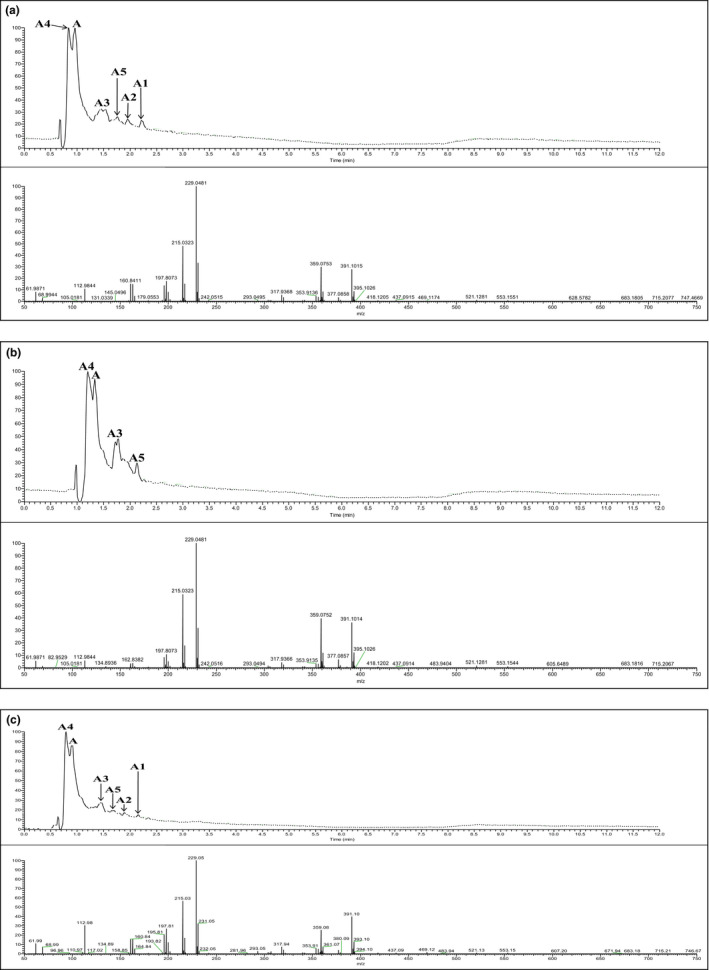
Major compounds identified using LCMS and their molecular profile

The major peaks of HPLC chromatograms and TICs (Figure 2) were tentatively identified as 2,3,5,6‐tetrachloro‐4‐(4‐phenylhepta‐1,2,5,6‐tetraen‐4‐yl) phenol (A‐A5). It was tentatively identified on the basis of monoisotopic mass and masses of its fragment ions. As shown in Figure 2, compound A had a monoisotopic mass of *m/z* 391. Therefore, the compound (2,3,5,6‐tetrachlorophenol) with a mass of *m/z* 229 was the basic unit of compound A. Similarly, the mass of fragment ions (A1–A5) were *m/z* 367, 359, 337, 323, 229, and 213, respectively. The major phenolic compound A and its fragments ions with molecular structures are presented in Figure 3. Further investigation of this phenolic compound (A) revealed that it was a type of phenolic derivative that was assumed to have formed from the hydrolysis of sugar solutions with a strong acid (HCl) during the extraction of polyphenols (Barrera, Betoret, & Seguí, 2020).

All flavonoids were detected in trace amounts. These flavonoids were represented exclusively by flavones (apigenin, luteolin, and tricin; Table 4). Although the HPLC peaks of these flavonoids were detectable, their concentrations were too low for quantification. Consequently, the concentrations of apigenin, luteolin, and tricin were detected but below the quantification limits. The results were in consistent with those of previous studies, which reported that flavones (apigenin, luteolin, and tricin) are found in sugarcane in low amounts (Colombo, Yariwake, Queiroz, Hdjoko, & Hostettmann, 2005; Duarte‐Almeida et al., 2011). MRBS contained the lowest concentrations of luteolin (but the results were not significant), but it had the highest amounts of apigenin. By contrast, BS contained the highest tricin content. Barrera et al. (2020) reported that tricin and apigenin are the most abundant phenolics in raw sugars. No significant differences in the concentrations of flavonoids were found among all samples. Other phenolic compounds were not detected in the sugar samples because they were probably lost during the extraction, especially during thermal degradation (Payet et al., 2006). Finally, the concentrations of inverted sugars in MRBS also affected the evaluation of phenolic and flavonoid contents (Seguí et al., 2015).

### 3.6 Antioxidant activities of sugar samples

The antioxidant activities of the sugar samples were determined via DPPH radical scavenging effect and FRAP assays. As shown in Table 3, RS had the lowest DPPH inhibition activity and FRAP value. The inhibition activity and FRAP value of MRBS were significantly higher than those of RS and BS (*p* < .05). RS had the lowest DPPH inhibition activity, followed by light BS, dark BS, and raw BS (Seguí et al., 2015). Unrefined sugars retain large amounts of phenolic and flavonoid compounds from sugarcane juice. The contents of phenolics, flavonoids, and other compounds are usually responsible for the dark coloration of sugars. No data on the antioxidant activity of branded unrefined sugars are available (Lee et al., 2018). Nevertheless, several studies have documented that unrefined sugars in local markets have higher antioxidant activities than RS (Asikin, Hirose, Tamaki, Oku, & Wada, 2016; Payet et al., 2005; Segi et al., 2015).

However, the DPPH inhibition activities of light and dark BS were not significantly different. A previous study reported that granulated RS (0.013) had FRAP values (mmol/130 g) lower than those of BS (0.516–0.986) and raw cane sugar (0.186) (Phillips et al., 2009). Raw cane sugar has a lower DPPH inhibition activity (22.1%) than all the samples studied (>88%); the inhibition activity of commercial BS ranges from 14.5% to 26.9% (Payet et al., 2005). Another study reported that raw cane sugar has a DPPH scavenging activity of 0.4–0.9 mmol Trolox equivalent/100 g sample and a FRAP value of 0.204 mmol/100 g (Jaffé, 2015). Thus, the antioxidant activity of the sugars is attributed to their polyphenolic constituents (mainly flavonoids, polyphenol, and phenolic acids) (Yao et al., 2004). The results for antioxidant activity were in good agreement with those of previous studies in terms of total phenols, flavonoids, and color values. For instance, tricin and apigenin are the most abundant phenolics in cane sugars. These phenolics are considered as an important bioactive constituent of foods and are postulated as nutraceuticals with antiproliferative and chemopreventive agents (Barrera et al., 2020). Several studies have proved that the correlation between total polyphenols and antioxidant activity are generally good but depends on the nature of the sample and the effects of other compounds on antioxidant activity (Guimaraes et al., 2007; Kadam et al., 2008; Moure et al., 2001). Barrera et al. (2020) found that the physicochemical and antioxidant properties of raw sugars are strongly related to the degree of refinement of each product. In the present study, total polyphenols and antioxidant activity were not significantly correlated. Pearson correlation coefficient analysis showed that the TPC of the sugar samples was not significantly correlated with DPPH inhibition. Aside from DPPH, the TPC of the sugar samples was strongly correlated with FRAP values (*r* = 0.981, *p* < .001). The TAC of the sugar samples was also strongly correlated with FRAP values (*r* = 0.826, *p* < .01). Payet et al. (2005) also demonstrated that the percentage of DPPH inhibition is not significantly correlated with TPC (*r* = 0.462, *p* > .05). By contrast, Feng, Luo, Zhang, Zhong, and Lu (2014) and Seguí et al. (2015) found that the percentage of DPPH inhibition is strongly and positively correlated with TPC (*r* = 0.881–0.996, *p* < .01). Thus, the present study demonstrated that antioxidant content had no correlation with DPPH inhibition activity. However, an increase in phenolic concentration would increase antioxidant activity.

### 3.7 Cytotoxic effects of sugar samples

Few data on the cytotoxic effects of natural sugars in vivo or ex vivo systems are available. Thus, this study aimed to determine the cytotoxicity of RS, MRBS, and BS. All sugar samples were not cytotoxic to 3T3‐L1 adipocytes (Table 5). The cells treated with sugar solutions of RS, BS, and MRBS had 100% viability. However, the cells treated with refined extracts had significantly lower cell viability than the BS extracts. These finding indicated that the sugar solutions were not cytotoxic to adipocytes. The polyphenols extracted from MRBS were also not cytotoxic to adipocytes, except for RS extract, which are potentially cytotoxic to 3T3‐L1 cells.

**Table 5:** Viability of 3T3‐L1 cells treated with sugar solutions and extracts of MRBS and other sugars for up to 72 hr

Sample	Cell viability (%)		
	24 hr	48 hr	72 hr
Control	100 ± 0.00	100 ± 0.00	100 ± 0.00
*Solution*			
Refined	100 ± 0.00	100 ± 0.00	100 ± 0.00
Brown	100 ± 0.00	100 ± 0.00	100 ± 0.00
MRBS	100 ± 0.00	100 ± 0.00	100 ± 0.00
*Extract*			
Refined	100 ± 0.00	100 ± 0.00	77.82 ± 10.55^b^
Brown	100 ± 0.00	100 ± 0.00	94.31 ± 13.09^a^
MRBS	100 ± 0.00	100 ± 0.00	100 ± 0.00^a^

Note

Different lowercase superscript letters (a, b) show a significant different between the sugar samples.

Vallie et al. (2012) demonstrated that concentrations of sugar cane and sugar beet molasses higher than 100 mg/ml can cause cell death, whereas concentrations up to 10 mg/ml have no cytotoxicity to HepG2 cells. The results of the present study were consistent with those of Vallie et al. (2012).

### 3.8 Effects of sugar solutions on hatching and survival rates

**Figure 4:**
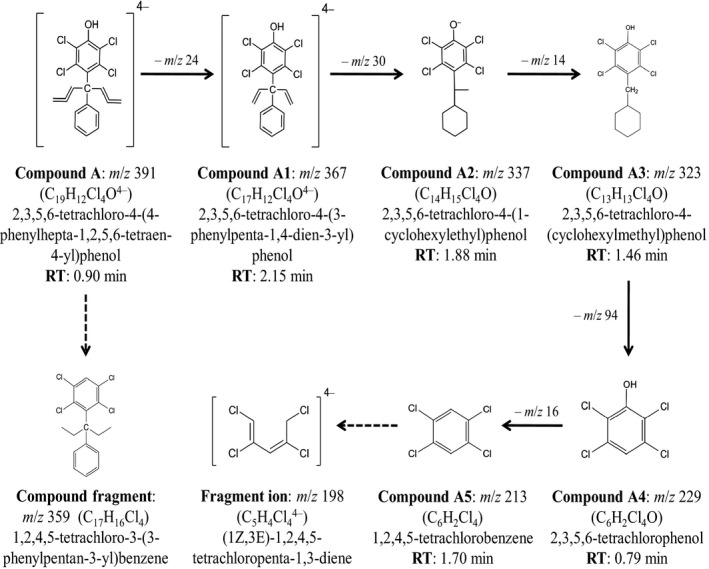
Hatching rates of zebrafish embryos treated with sugar solutions of (A) refined sugar, (B) brown sugar and (C) MRBS. *C: control (paracetamol)

**Figure 5:**
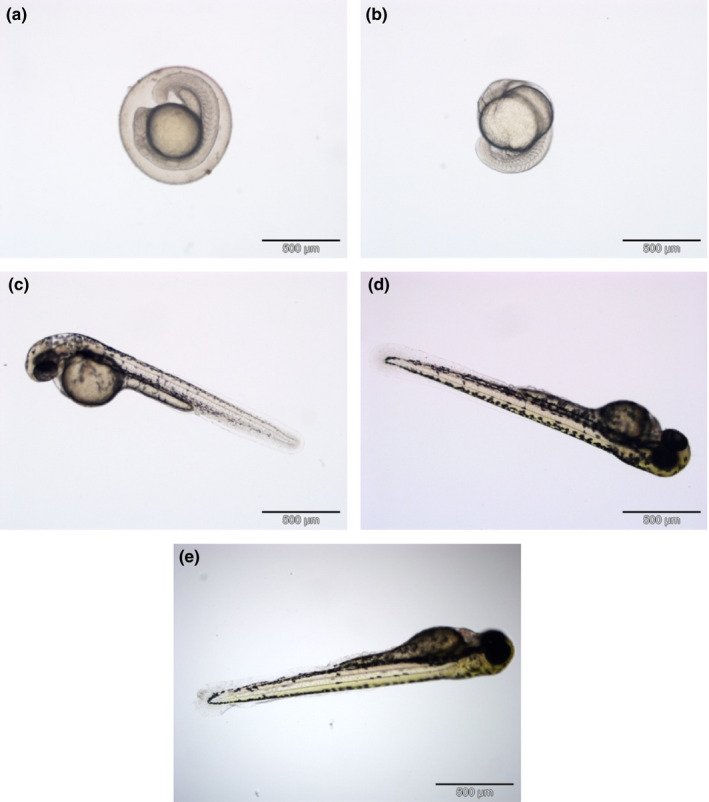
Survival rates of zebrafish embryos treated with sugar solutions of (A) refined sugar, (B) brown sugar and (C) MRBS. *C: control (paracetamol)

As shown in Figure 4a, all embryos treated with RS solution hatched at 96 hr of incubation (as indicated by the light blue color in the curve). Compared with the control (paracetamol), most concentrations delayed hatching, except 2.5 mg/ml. Similar to control (paracetamol), all embryos treated with 2.5 mg/ml sugar concentration hatched at 72 hr. The survival rate of the embryos treated with RS solution at all concentrations was 100%, except for the embryos treated with 2.5–10 mg/ml sugar solution (Figure 5a). At 2.5–10 mg/ml solution concentrations, only 80% of the embryos survived. LD_50_ was not tabulated because no toxicity was observed in the highest concentration tested. All embryos hatched when treated with all BS solution concentrations (Figure 4b), except for 2.5 and 10 mg/ml. Compared with the control, most concentrations delayed hatching.

As shown in Figure 5b, the survival rate of the embryos treated with BS solution was similar to that treated with RS solution, except at 10 mg/ml solution concentration. At 10 mg/ml of BS solution, the survival rate of the embryos at 96 hr was 70%, 10% lower than the survival rate at 72 hr. Similarly, no LD_50_ value was obtained for BS solution.

After 96 hr of incubation, all embryos treated with MRBS solution hatched, except for the embryos treated with sugar solution at concentrations from 2.5 mg/ml to 10 mg/ml (Figure 4c). Like the embryos treated with BS, only 80% of the embryos hatched at 96 hr of incubation when treated with MRBS solution at concentrations of 2.5 and 10 mg/ml. Compared with the control (paracetamol), most concentrations delayed.

The survival rate of zebrafish embryos treated with MRBS solution was better than that of embryos treated with BS (Figure 5c). The survival rate of the embryos treated with MRBS at the solution concentration of 10 mg/ml for 96 hr was significantly higher (80%) than that of the embryos treated with BS (70%). However, the embryos treated with both sugar solutions had a high survival rate, demonstrating that both types of BS did not affect the survival rate of (or were not toxic to) zebrafish embryos.

### 3.9 Effects of sugar solutions on heart rate

**Figure 6:**
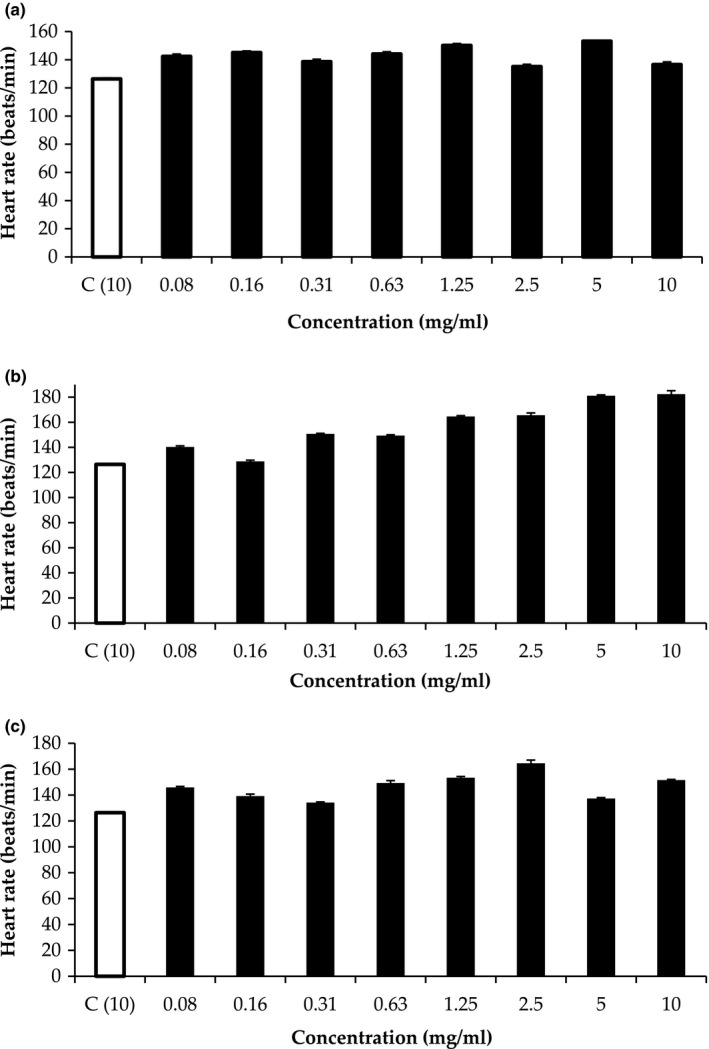
Heart rates of zebrafish embryos treated with sugar solutions of (A) refined sugar, (B) brown sugar and (C) MRBS. *C: control (paracetamol)

The heart rate of zebrafish embryos ranged from 120 beats/min to 180 beats/min (Figure 6). Heart rates were measured by direct visual examination of ventricles as they beat. Embryos of the negative control group had heart rates of 120–130 beats/min (Chan, Lin, & Cheng, 2009). Results showed that the heart rate of zebrafish embryos decreased due to treatment with RS solution at concentrations between 2.5 and 10.0 mg/ml (Figure 6a). However, the embryos treated with paracetamol had slightly lower heartbeat (beats/min), indicating that the RS solution slightly increased the heart rate of zebrafish embryos.

As shown in Figure 6, treatment with MRBS and BS solutions also slightly increased the heart rate of the embryos. The heart rate of the embryos increased as the concentration of BS solution increased. At the same concentration of 10 mg/ml, the embryos treated with paracetamol had significantly a lower heart rate (beats/min) than the embryos treated with BS solution. These results demonstrated that the BS solution increased the heart rate of the zebrafish embryos because BS had high antioxidant properties.

By contrast, treatment with MRBS only slightly reduced heart rate as solution concentration increased. Although the heart rate of embryos increased as the concentration of MRBS increased from 0.31 to 2.5 mg/ml, the heart rate significantly decreased at the sugar concentration of 5 mg/ml and only slightly increased at the sugar concentration of 10 mg/ml. Kim et al. (2015) reported that phenolic compounds may affect the heartbeat of aquatic fish. For instance, they have found that phlorotannin isolated from a brown alga considerably decreased the rate heart of zebrafish embryos treated with 150 mM glucose. In the present study, the phytochemicals in MRBS, such as 4‐hydroxybenzoic acid, chlorogenic acid, and protocatechuic acid, and trans‐Ferulic acid, within the concentration range of 5–10 mg/ml, decreased the heart rate of the zebrafish embryos compared with BS.

## 4 CONCLUSION

BS, especially MRBS, had fair amounts of antioxidants. The minimal refining process of MRBS retained some of its phytochemicals, vitamins, and minerals. RS had lower amounts of nutrients than BS, except for sucrose. Heavy sugar refinement or processing causes a considerable decrease in antioxidants (polyphenols) in sugarcane. Minimal processing of BS retains some of its phytochemicals, such as phenolic acids and flavonoids. This study suggested that small amounts of phenolics in BS pose no threat to humans. This result was further confirmed by in vitro and in vivo assays. Total polyphenols and antioxidant activity were not significantly correlated. Overall, MRBS had a better nutritional quality in terms of physicochemical characteristics than the two other sugar samples. Moreover, MRBS also contained selenium, which is not found in many types of sweeteners. These antioxidants and nutrients found in BS, especially in MRBS, may provide extra benefits compared with RS. However, the amounts of these antioxidants and nutrients were lower in MRBS than those in other sugar alternatives. Therefore, future studies should investigate the antidiabetic effects of MRBS and assess the efficacy of polyphenols in preventing metabolic‐related diseases.

## CONFLICT OF INTEREST

The authors declare that they have no known competing financial interests or personal relationships that could have appeared to influence the work reported in this paper.

## Author Contributions

Conceptualization, A.A., H.E.K.; methodology, A.A., A.B.S.; formal analysis, H.E.K., N.A.A.A.K; writing original draft preparation, S.S, B.N.M.Y..; writing—review and editing, A.A.; supervision, A.A.; project administration, A.A., H.E.K.; funding acquisition, M.Z., B.N.M.Y.
